# Arthroscopic surgery for scaphoid nonunion: a 10-year systematic literature review

**DOI:** 10.1007/s12306-023-00805-x

**Published:** 2024-02-10

**Authors:** Alberto Rinaldi, Federico Pilla, Ilaria Chiaramonte, Davide Pederiva, Fabio Vita, Francesco Schilardi, Andrea Gennaro, Cesare Faldini

**Affiliations:** Present Address: 1St Orthopaedics and Traumatology Clinic, IRCCS Istituto Ortopedico Rizzoli, via Giulio Cesare Pupilli, 1, 40136 Bologna, Italy

**Keywords:** Arthroscopic, Nonunion, Pseudoarthrosis, Scaphoid

## Abstract

The purpose of the study was to investigate whether arthroscopic treatment of carpal scaphoid nonunions by osteosynthesis with bone grafting represents a successful surgical technique. This systematic literature review, conducted following the PRISMA guidelines, explores the past 10 years of clinical studies concerning the arthroscopic treatment of scaphoid nonunions. The most relevant keywords were used to search the databases, and the Downs and Black 27-item checklist has been used as quality assessment tool. Twelve papers that meet the premised eligibility criteria have been identified. These studies demonstrate the efficacy of this surgical solution, achieving a postoperative union rate of 96% in the average time of 13.5 weeks. Regardless of the method of synthesis and the origin of the graft used, excellent results were obtained. Patients who underwent this procedure reported a pain reduction of almost 80% compared to the preoperative level, improvement in grip strength close to 40%, and recovery in wrist function during daily activities. Arthroscopy has numerous advantages compared to the open approach. These are technically recognized by the surgeon and by the patient. Some disadvantages include a longer intraoperative time and considerable significant technical difficulty. Arthroscopic treatment of scaphoid nonunion by osteosynthesis with bone graft achieves a 96% union rate of the treated scaphoid with satisfying clinical results.

## Introduction

The scaphoid is proven to be the most commonly fractured carpal bone. It represents 2.4% of all wrist fractures and accounts for approximately 60%–80% of carpal fractures. Annually, this occurs in 23–43 per 100,000 people [1]. A major part of scaphoid fractures occurs in male adolescents and young adults, high-function-demand patients [2, 3].

Despite an adequate non-surgical treatment, around 10%–15% of these fractures do not heal [4].

A bone nonunion is defined as a fracture that has failed to heal within the first 9 months following injury with no signs of healing for at least 3 consecutive months [5]. The risk of nonunion increases with delayed diagnosis, inadequate immobilization, fracture instability, fracture displacement, and associated ligamentous injury [6]. The correct diagnosis and treatment for these conditions are crucial to avoid the natural history of untreated scaphoid nonunion. This is known as a scaphoid nonunion advanced collapse (SNAC) and leads to progressive arthritis. There is a possibility that this exclusively involves the scaphoid bone (sclerosis, cyst formation, and resorptive changes), the radioscaphoid joint, or all of the wrist [7].

The treatment strategies for scaphoid nonunion include rigid fixation, using both screws and Kirschner wires, alone or in conjunction with bone grafting.

In 2002, a systematic review concluded that in unstable nonunions, screw fixation with grafting was superior to K-wires and wedge grafting (94% vs 77% union rate, respectively) [8]. In 2015, a more recent one established that current evidence did not demonstrate a significantly superior method for the treatment of scaphoid nonunion [9]. However, this still remains an open question.

Nowadays, the use of arthroscopy in wrist surgery is increasingly widespread. It holds a definite role in managing difficult scaphoid fracture, delayed union and nonunion, providing a thorough wrist assessment, a comprehensive approach for scaphoid fracture and its sequelae in a minimally invasive manner, a favorable biological environment for the fracture union, and minimal surgical trauma to the ligamentous architecture and vascularity [10]^.^

However, the technical difficulties, cost of instruments, longer time required for surgeries, and the long lengthy learning curve needed for the safety management of this technique, determine that the approach of open surgery remains the most widely used method for scaphoid nonunion fixation.

Since the last review concerning the arthroscopic management of scaphoid nonunion has been performed several years ago [11], in light of the considerable number of studies published in recent years, the aim of this article is to investigate whether the arthroscopic approach in scaphoid nonunion is a successful surgical solution to achieve both clinical and radiological valuable results.

## Materials and methods

This current work was carried out in accordance with Preferential Reporting Items for Systematic Reviews and Meta-Analyses (PRISMA) guidelines.

### Eligibility criteria

We conducted a systematic screening of the available literature from January 1, 2012, to December 31, 2022, searching for studies dealing with arthroscopic treatment for scaphoid nonunion. All surgical techniques associated with the utilization of wrist arthroscopy were incorporated, even when compared to other surgical approaches. Arthroscopic management of acute scaphoid fracture was not considered. We selected all the studies where results were expressed from a clinical and radiological point of view, excluding findings based on imaging alone.

The types of study considered for inclusion were randomized controlled trials (RCTs), retrospective studies (RS), retrospective case series (RCS), and prospective cohort studies (PCS). Case reports, literature reviews, meta-analyses, technical notes, instructional courses, biomechanical and/or in vitro studies, and cadaver experiments were excluded.

Only peer-reviewed publications, written in English, were considered.

### Information sources and search strategy

The literature review was carried out in January 2023. The search terms included “arthroscopic,” “non-union” or “nonunion,” “pseudoarthrosis,” and “scaphoid.” The online databases used for the research are PubMed-MEDLINE, Google Scholar, and Cochrane Library (central trials database). Two reviewers independently performed the investigation (A.R. and I.C.).

### Selection process and data collection

An initial screening based on the title and abstract was performed. The studies eligible for inclusion were further investigated by obtaining the full text for a complete evaluation. The bibliography of the most relevant articles had been checked to identify potentially missed eligible papers. The complete process of selection had been summarized as follows according to the PRISMA guidelines.

For every paper, we summarized the most relevant parameters, as shown in Table 1.

**Table 1:** Available studies concerning the arthroscopic treatment in scaphoid nonunion according to the inclusion criteria

Study	Type of study	No. of patients	Mean FU duration	Average union time	Union rate	Fixation technique	Bone graft	Clinical evaluation parameters
Waitayawinyu et al. [14]	RS	22	32.5 mths	15, 3 wks	100%	Screw	Autologous olecranon	VAS score for pain; ROM; grip strength; MMWS; DASH score; MCIDs of DASH
Lee et al. [15]	RS	15	30 mths	9, 7 wks	100%	K-wire	Autologous iliac crest	ROM; grip strength; MMWS; VAS for pain
Wu et al. [16]	RS	20	31 mths	14 wks	100%	K-wire (12) screw (6) K-wire + screw (2)	Autologous iliac crest	Grip strength; patient-reported outcome measures; ROM
Hsiung et al. [17]	RS	41	38.1 mths	4, 6 mths	92.6%	Screw	Autologous distal radius	VAS for pain; ROM; grip strength
Wang et al. [18]	RS	21	31.3 mths	16, 3 wks	90.5%	Screw	Autologous 8 iliac crest 13 distal radius	DASH; ROM; grip strength; VAS for pain
2019 Wong et al. [19]	RS	125	34 mths	14 wks	90.3%	Screw (38) K-wire (87)	Autologous iliac crest	ROM; grip strength; wrist function performance score; VAS for pain; return to work status
Liu et al. [20]	RS	25	21 mths	Stable fx: 11 wks unstable fx: 13 wks	100%	Screw	Autologous iliac crest or distal radius	ROM; MWS; PRWE
Lee et al. [21]	RS	27	18 mths	10 wks	96%	K-wire	Autologous iliac crest	ROM; pinch strength; grip strength; MWS; VAS for pain
Oh et al. [22]	RCoS	28 art 34 open	39.6 mths	Not specified	96.4–97.1%	Screw	Not specified	VAS for pain; ROM; grip strength; MWS; DASH
Cognet et al. [23]	PS	23	17.3 mths	4 mths	100%	Various techniques with screw and K-wire	Autologous distal radius	VAS for pain; ROM; grip strength
Kang et al. [24]	RS	33	33 mths	8–10 wks	97%	Screw	Autologous iliac crest	VAS for pain; grip strength; ROM; MWS; DASH
Kim et al. [25]	RS	36	37 mths	11 wks	86%	Screw	Autologous distal radius 16 patients (44%)	ROM; grip strength; DASH; PRWE; MMWS

*N°*  Number; *FU* Follow-up; RS: retrospective study; *RCoS* Retrospective comparative study; *PS* Prospective study; *mths* Months; *wks* Weeks; *art* Arthroscopic; *open* Open surgery; *fx* Fracture; *VAS* Visual analog scale; *MMWS* Mayo modified wrist score; *MWS* Mayo wrist score; *DASH* Disability of the arm, shoulder, and hand score; *MCID* Minimal clinically important difference; *ROM* Range of motion; *PRWE* Patient-rated wrist evaluation score

### Quality assessment

The selected articles were investigated by a quality assessment tool. In relation to the type of studies that emerged and their different characteristics, the appropriate tool was identified in the Downs and Black (1998) 27-item checklist [12]. As done by Trac et al. [13], the last item was modified. The tool was used by two independent reviewers (D.P. and A.R.).

## Results

### Study selection and characteristics

**Fig. 1:**
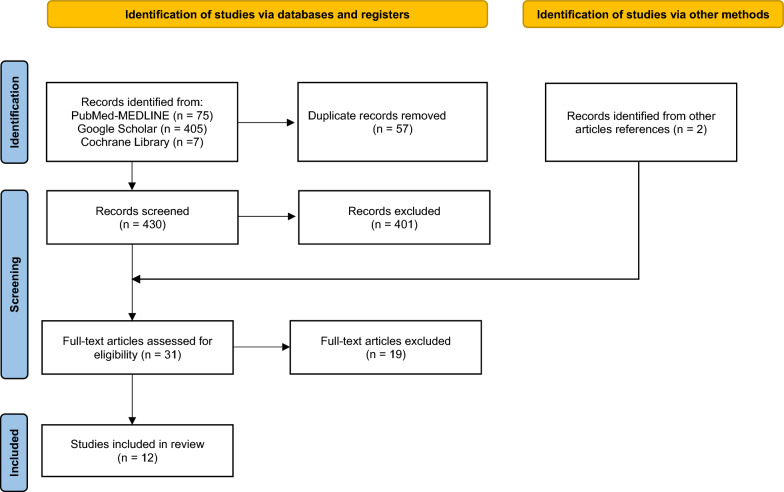
PRISMA flow diagram for systematic reviews that included search strategy and number of records analyzed

The study selection process is summarized in Fig. 1, using the PRISMA flowchart.

In Table 1, the most important characteristics are presented.

### Study quality assessment

The quality assessment results are reported in Table 2.

**Table 2:** Quality assessment results according to Downs and Black 27-item checklist

Study	Waitayawinyu et al. [14]	Lee et al. [15]	Wu et al. [16]	Hsiung et al. [17]	Wang et al. [18]	Wong et al. [19]
Result	Good (21)	Poor (14)	Fair (18)	Good (20)	Fair (17)	Poor (12)

Study	Liu et al. [20]	Lee et al. [21]	Oh et al. [22]	Cognet et al. [23]	Kang et al. [24]	Kimet al. [25]
Result	Fair (16)	Fair (16)	Good (22)	Poor (10)	Fair (17)	Fair (18)

### Cohort characteristics

The number of patients enrolled averaged 35 patients (range 15–125, SD 29 IQR 14), with a mean follow-up of 30.2 months (range 17.3–39.6, SD 7.5 IQR 7).

In most cases, the subjects included in the studies are symptomatic patients with delayed union or unstable scaphoid nonunion. Delayed union is specified as radiological evidence of a permanent fracture beyond 8 weeks after injury. In the majority of cases, nonunion is defined as radiological evidence of a permanent fracture with the presence of sclerosis, cystic lesions, or lack of substance greater than 2 mm objectifiable on imaging, at least 6 months after the event of fracture. Some authors have considered additional radiographic criteria, regarding unstable scaphoid nonunion [22, 25].

The most common exclusion criteria are stable nonunion without substantial bone loss, stable fibrous union, wrist comorbidities (osteoarthritis or radio-carpal instability, other ipsilateral distal radius or carpal fractures), avascular necrosis of the proximal fragment, advanced grade SNAC, inadequate follow-up, and revision surgery.

Some studies included patients with stage I SNAC [15, 18, 19, 21] and radio-carpal or midcarpal osteoarthritis [21], with imaging suggestive of avascular necrosis [21]. One author [19] considered nonunion to be a permanent fracture at 3 months instead of 6 and described revision surgery, SNAC grade II, and avascular necrosis of the proximal pole.

Specific exclusion criteria are reported for humpback deformity and DISI [24] and for a study concerning only proximal pole fractures [16].

Associated injuries (TFCC, lunotriquetral ligament, and scapholunate ligament) were not considered to be reasons for exclusion, besides one study that excluded patients with scapholunate ligament injury [17].

### Surgical technique

All selected studies describe surgeries performed with arthroscopic technique. In one case, it is compared with the open technique [22].

They were performed using cannulated headless screws (7), Kirschner wires (2), or a miscellany of both techniques (3). Autologous bone grafts, harvested from iliac crest (5), distal radius (3), olecranon (1), or multiple targets (1), were used in all studies. One article did not specify the harvest site [22].

The average duration of the surgical operation, where specified, was reported to take 131 min (range 83–213, IQR 90 SD 52).

### Radiographic and clinical outcomes

Seven authors performed postoperative CT scans as routine to confirm union, and three used CT to better study doubtful cases [15, 17, 25]. One author did not use CT in any patient to confirm union [21]. There is less consensus about preoperative imaging, where only two authors systematically used CT [20, 24].

Clinical evaluation parameters are meticulously listed in Table 1. The most used are VAS for pain, wrist ROM, grip strength, Mayo wrist score, and DASH score.

The union rate obtained in the different studies averaged 96% (range 88–100%, IQR 7.5% SD 5%). The medium time for achieving this result took 13.5 weeks (range 8–20 weeks, IQR 5.3 SD 3.5). Some authors have performed additional radiological measurements [15, 18, 22, 25]. The most used radiological parameters were SLA (scapholunate angle) and RLA (radiolunate angle). All of the authors established a statistically significant improvement in SLA following surgery. With the RLA, the studies that had been evaluated returned uneven results. Kim [25] and Oh [22] deepened the radiographic investigation by evaluating multiple additional parameters, which overall improved following surgery. Despite this, no correlation was found between them and the clinical outcome.

From the clinical point of view, in all of the studies, the level of pain achieved a statistically significant improvement, as measured by VAS, which decreased on average by 79%, from 5.2 to 1.1 with a reduction of 4.1 points (range 3.0–5.7, IQR 1.2 SD 1.0).

Each author reports a gain in strength: The average increase is 39.8% (range 11.6–157.3% IQR 19.8% SD 40.4%).

Clinical scoring systems (Mayo wrist score, DASH, etc.) were increased overall in all studies in which they were used to evaluate the surgical outcome. Wrist range of motion (ROM), assessed in 11 studies, improved significatively in seven of them.

## Discussion

### Patient characteristics

The average number of 35 patients is always the result of collecting cases treated over several years, even in different centers [20], a time frame necessary to achieve a significant number. Despite this, authors reported that a larger number of cases selected with the same specifications would be desirable to enhance the statistical [14–16, 18, 20, 21, 23, 25].

The study design, which is retrospective in 11 of 12 cases, could overestimate the benefits of the surgical treatment, as the selection of patient inclusion criteria carried out may be affected by selection bias [24].

### Clinical outcomes

The most evident result is the unanimous clinical benefit reported by the authors in the totality of the selected studies.

Patients have reported a decrease of nearly 80% in wrist pain at the last follow-up compared to what had been declared preoperatively. The results reflect an enormous therapeutic success positively impacting physical and psychological well-being, daily life activities, employment, social, family and health system fields, and the direct and indirect related costs [26].

The clinical impact is equally evident from the point of view of the scoring systems used to detect the impact of the disease of specific wrist function and its extension to the overall level of the patient’s disability. The statistically significant increase in the evaluating systems in all studies is unequivocal evidence of the benefit gained. Another leading cause that is detected in all studies is the recovery of grip strength, with an average value close to 40%.

Regarding the variation with the range of motion of the operated wrist, statistically, it improved significantly in seven out of 11 studies where it was calculated.

One of the most dissimilar criteria is the inclusion or exclusion of patients with stage I SNAC. Considering there are excellent scaphoid union results in studies where patients with this condition were treated [15, 18, 19, 21], which do not differ in any way from papers in which this condition was found to be a reason for exclusion, it can be deduced that the techniques described are appropriate and successful even in patients with stage I SNAC.

### Radiological outcomes and imaging strategies

The unions were confirmed with a success rate of 96%.

Additional radiologic parameters collected by some authors on the recovery of carpal bone alignment yielded concordant results solely on the normalization of SLA. Previously, some authors have suggested that there is a correlation between the significance of residual deformity and the clinical outcomes of the surgical treatment of scaphoid fractures [27]; others, however, have not identified a solid association between the two entities [28, 29]. The articles included in this review are unable to provide an answer to this dilemma.

The methodology with the lowest rate of bias is the confirmation by CT scan analyzed by a blind doctor who was not involved in the surgical process. The use of CT as a diagnostic test for confirmation of union appears to be widely agreed upon by author. Lee [21], the only author who did not use CT, reported this peculiarity as a significant limitation.

The systematic preoperative MRI was performed by only one author [18]. The previous studies have shown that MRI with contrast has failed to provide a correlation between vascularization and the presence of intraoperative punctate bleeding of the fragments [30, 31].

### Surgical techniques

The results demonstrate an excellent success rate regardless of the surgical techniques used. This conclusion agrees with what has been previously reported in the available scientific literature: An excellent systematic review carried out in 2015 by Pinder et al. [9], revealed that current evidence does not demonstrate a significantly superior method for the treatment of scaphoid nonunion. Vascularized and nonvascularized bone grafts had a similar union incidence, distal radius and iliac crest bone grafts had similar union rates, screw and K-wire fixation had a similar success.

Though the intention of this review is not to compare fixation methods, types of grafts used, and site of harvesting, the excellent union rates achieved with each type of synthesis method and from any site of graft harvesting yet unequivocally parallel what has been previously described by colleagues, suggesting that the technique to be used should be sought in the surgeon's confidence with one approach compared to others.

Regardless of the method used, the maintenance of the vascular support of the scaphoid, the preparation of the graft site by accurate debridement of the fibrous tissues and the most superficial necrotic bone of the fracture site, the reduction of the bone gap, the affixing of bone grafting, and the internal stabilization are remarkably important [21, 23].

### Surgical complications

The perioperative complications following wrist arthroscopy ranging from 1.2% to 5% and include infections, extensor tendon injury, superficial sensory neuropraxia, dorsal wrist ganglion, stiffness, or complex regional pain syndrome [24]. In this selection of papers, exclusively Wong [24] reports the occurrence of some perioperative complications. According to other authors [22, 24], such the small number of reported adverse events is due in part to the low complication rate that accompanies this type of surgery. On the other hand, the small number of cases selected limits the statistical potentiality to evaluate rare events.

### Wrist arthroscopy

Wrist arthroscopy presents a considerable learning difficulty, but advantages, compared to classical techniques, lead an increasing number of surgeons to use arthroscopic techniques. It is a relatively safe procedure that in current day represents the diagnostic and therapeutic gold standard for an extensive variety of pathological conditions.

In the treatment of scaphoid nonunion, this technique allows direct evaluation of the nonunion and other associated soft tissue lesions, if present, with the possibility of intervention and their eventual repair [15, 18, 21, 24]; it facilitates the identification with the correct point of entry of the guide wire and the fixation hardware [24]; it preserves the precarious vascularization of the scaphoid while sparing adjacent tissues [14–16, 18, 21–24]; it allows an excellent debridement of the bone graft site [23]; it avoids wrist and carpal ligaments injury as it does not require an open arthrotomy, which may result in carpal malalignment [14, 16, 20] through a compromised mechanical strength and impaired proprioceptive and neuromuscular control; compared with open surgery, pain, stiffness, and scarring are limited [15].

Arthroscopy also demonstrates its usefulness in the treatment of coexisting intra-articular disorders, which are reported between 42 and 83% [21, 24, 32–34], such as TFCC tears, SL ligament injuries, and LT ligament injuries.

The limitations of using arthroscopic treatment of scaphoid nonunion are the more stringent exclusion criteria of patients with comorbidities at the wrist and carpus (osteoarthritis or radio-carpal instability, concomitant distal radius or carpal fractures, and advanced grade SNAC), severe comminution or loss of substance of the scaphoid [23], the longer learning process of the technique [22], and the longer time required for surgery.

The average cost, where estimated, of the operating room, shows no significant difference from that of the same surgery performed in open [22].

### Limitations of the evidence

These results should be further corroborated in the future by the creation of multicenter studies with the same inclusion/exclusion criteria and patient evaluation scores, in order to homogenize the subject cohort, reduce selection and allocation biases, and reach a larger number of cases to enhance the statistical significance of the research.
